# Development and internal validation of a clinical prediction model for serious complications after emergency laparotomy

**DOI:** 10.1007/s00068-023-02351-4

**Published:** 2023-08-31

**Authors:** Stamatios Kokkinakis, Evangelos I. Kritsotakis, Konstantinos Paterakis, Garyfallia-Apostolia Karali, Vironas Malikides, Anna Kyprianou, Melina Papalexandraki, Charalampos S. Anastasiadis, Odysseas Zoras, Nikolas Drakos, Ioannis Kehagias, Dimitrios Kehagias, Nikolaos Gouvas, Georgios Kokkinos, Ioanna Pozotou, Panayiotis Papatheodorou, Kyriakos Frantzeskou, Dimitrios Schizas, Athanasios Syllaios, Ifaistion M. Palios, Konstantinos Nastos, Markos Perdikaris, Nikolaos V. Michalopoulos, Ioannis Margaris, Evangelos Lolis, Georgia Dimopoulou, Dimitrios Panagiotou, Vasiliki Nikolaou, Georgios K. Glantzounis, George Pappas-Gogos, Kostas Tepelenis, Georgios Zacharioudakis, Savvas Tsaramanidis, Ioannis Patsarikas, Georgios Stylianidis, Georgios Giannos, Michail Karanikas, Konstantinia Kofina, Markos Markou, Emmanuel Chrysos, Konstantinos Lasithiotakis

**Affiliations:** 1 Department of General Surgery, School of Medicine, University Hospital of Heraklion, University of Crete, Heraklion, Greece; 2 Laboratory of Biostatistics, School of Medicine, University of Crete, 71003 Heraklion, Crete Greece; 3 Department of Surgical Oncology, School of Medicine, University Hospital of Heraklion, University of Crete, Heraklion, Greece; 4 Department of Surgery, School of Medicine, University General Hospital of Patras, University of Patras, Patras, Greece; 5 Department of Surgery, School of Medicine, General Hospital of Nicosia, University of Cyprus, Nicosia, Cyprus; 6 First Department of Surgery, Laikon General Hospital, National and Kapodistrian University of Athens, Athens, Greece; 7 Second Propaedeutic Department of Surgery, Laikon General Hospital, National and Kapodistrian University of Athens, Athens, Greece; 8 Department of Surgery, School of Medicine, University General Hospital Attikon, University of Athens, Athens, Greece; 9 Department of Surgery, General Hospital of Volos, Volos, Greece; 10 Department of Surgery, General Hospital of Trikala, Trikala, Greece; 11 Department of Surgery, University Hospital of Ioannina, Ioannina, Greece; 12 Department of Surgery, School of Medicine, Ippokrateion General Hospital of Thessaloniki, Aristotle University of Thessaloniki, Thessaloniki, Greece; 13 Second Department of Surgery, Evangelismos General Hospital, Athens, Greece; 14 Department of Surgery, School of Medicine, University General Hospital of Alexandroupolis, University of Thrace, Alexandroupolis, Greece

**Keywords:** Prediction, Prognosis, Laparotomy, Validation, Complications

## Abstract

**Purpose:**

Emergency laparotomy (EL) is a common operation with high risk for postoperative complications, thereby requiring accurate risk stratification to manage vulnerable patients optimally. We developed and internally validated a predictive model of serious complications after EL.

**Methods:**

Data for eleven carefully selected candidate predictors of 30-day postoperative complications (Clavien-Dindo grade >  = 3) were extracted from the HELAS cohort of EL patients in 11 centres in Greece and Cyprus. Logistic regression with Least Absolute Shrinkage and Selection Operator (LASSO) was applied for model development. Discrimination and calibration measures were estimated and clinical utility was explored with decision curve analysis (DCA). Reproducibility and heterogeneity were examined with Bootstrap-based internal validation and Internal–External Cross-Validation. The American College of Surgeons National Surgical Quality Improvement Program’s (ACS-NSQIP) model was applied to the same cohort to establish a benchmark for the new model.

**Results:**

From data on 633 eligible patients (175 complication events), the SErious complications After Laparotomy (SEAL) model was developed with 6 predictors (preoperative albumin, blood urea nitrogen, American Society of Anaesthesiology score, sepsis or septic shock, dependent functional status, and ascites). SEAL had good discriminative ability (optimism-corrected c-statistic: 0.80, 95% confidence interval [CI] 0.79–0.81), calibration (optimism-corrected calibration slope: 1.01, 95% CI 0.99–1.03) and overall fit (scaled Brier score: 25.1%, 95% CI 24.1–26.1%). SEAL compared favourably with ACS-NSQIP in all metrics, including DCA across multiple risk thresholds.

**Conclusion:**

SEAL is a simple and promising model for individualized risk predictions of serious complications after EL. Future external validations should appraise SEAL’s transportability across diverse settings.

**Supplementary Information:**

The online version contains supplementary material available at 10.1007/s00068-023-02351-4.

## Introduction

Emergency laparotomy (EL) encompasses a broad spectrum of surgical procedures for various abdominal emergencies. Even in western healthcare systems, significant mortality and morbidity are EL patients' common denominator, with major postoperative complications affecting 24–47% of patients requiring EL globally [1–3]. The National Emergency Laparotomy Audit (NELA) in the UK recently noted a high 30-day mortality rate in EL patients (9.2%) and a prolonged length of stay (LOS) amongst survivors (median, 10 days) [4]. LOS prolongation was reported by NELA to reach a median of 15 days (IQR: 9–26) for high-risk patients (mortality risk > 5%) [4], which is indicative not only of the clinical burden but also of the substantial financial costs induced to healthcare systems from complicated EL cases.

Serious postoperative complications are more commonly reported in the literature as those with grade III or greater in the Clavien-Dindo classification, including complications that require surgical, endoscopic or radiological intervention, single or multiple organ dysfunction necessitating transfer to the Intensive Care Unit (ICU) and death [5]. This definition signifies a hard endpoint for emergency surgical patients and the ability to accurately risk stratify the patients for this endpoint may impact on advanced care practices and infrastructure by guiding assessments of the needs for critical care facilities, experienced endoscopists and interventional radiologists in high-risk general surgery patients [6]. Existing risk prediction models focus on mortality or overall morbidity after EL, as do the NELA model and the Predictive OpTimal Trees in Emergency Surgery Risk (POTTER) model [7, 8]. The American College of Surgeons National Surgical Quality Improvement Program (ACS-NSQIP) has proposed a prognostic model offering risk predictions for multiple outcomes across various surgical subspecialties, including the risk of serious complications [9]. However, the definition of serious postoperative complications in ACS-NSQIP refers to a group of major complications that is more diverse and broad than the widely accepted criterion of Clavien-Dindo grade III or greater, and this may hamper applicability across different settings.

Herein, we report on our effort to develop and internally validate a prognostic model to predict the risk of serious postoperative complications after EL based on the Clavien-Dindo grade and benchmark the performance of the new model against the rival ACS-NSQIP in a multicentre cohort of Greek patients.

## Methods

### Data source

The study was based on the HELAS multicentre cohort of patients who underwent EL in 11 centres in Greece and Cyprus (1 secondary and 10 tertiary-care hospitals), between 01.2020 and 05.2021 [10]. Inclusion and exclusion criteria in HELAS were identical to those of NELA (Supplementary Table S1). Patients were followed up until the 30th postoperative day.

### Reporting

The study complies with the Transparent Reporting of a Multivariable Prediction Model for Individual Prognosis or Diagnosis (TRIPOD) statement [11]. A TRIPOD checklist is included as Supplementary Table S2.

### Outcome

The outcome of interest to our prediction model was serious complications occurring up to 30 days after EL and classified as grade III or greater according to the Clavien-Dindo classification, including any postoperative adverse event requiring surgical, endoscopic or radiological intervention, organ dysfunction leading to ICU admission or death [5]. Whenever required, a post-discharge appointment with patients was scheduled on or after the 30th postoperative day to assess occurrence of complications.

### Predictors

Candidate predictors for model development were carefully selected based on literature review [12]. As little is known about predictors for serious complications after laparotomy, our selection of predictors was guided by a recent systematic review of 22 studies focusing on mortality after EL [13], and individual studies focusing on risk factors for adverse events in emergency abdominal surgery [14, 15]. The following preoperative variables were considered candidate predictors in our analysis: age, serum albumin level, white blood cell (WBC) count, blood urea nitrogen (BUN), American Society of Anaesthesiologists (ASA) score, dependent preoperative functional status, use of steroids, presence of disseminated cancer, preoperative dyspnoea, sepsis or septic shock, and ascites. Sepsis and septic shock were defined using the Sepsis-3 criteria [16], within 48 h before surgery. Dependent functional status was defined as requirement of partial or total assistance during 30 days before surgery. Use of steroids was defined as regular administration of oral or parenteral corticosteroid medication for a chronic medical condition, within the 30 days prior to surgery. Ascites was defined as presence of ascetic fluid within 30 days before surgery, documented either clinically or by imaging. Laboratory biomarker values (albumin, WBC, BUN) were chosen as those closest to the time when the decision to proceed with surgery was taken.

### Sample size

The HELAS cohort patients were not primarily recruited for developing a predictive model of serious complications. Therefore, the number of eligible patients in HELAS determined the sample size available for this study. However, we considered sample size and other requirements to ensure reliable model estimation and minimize the risk of producing an overfitted model that would be too much tailored to our development sample. As recommended by a commonly cited rule-of-thumb, the study included more than 100 complication events and 100 non-events and maintained an Event-Per-Variable (EPV) ratio greater than 10 [12]. In addition, penalized regression techniques were employed, that are expected to mitigate overfitting and improve prediction accuracy even when the EPV ratio is relatively small [17].

### Missing data

Missing values ranged between 0% and 1.3% for all candidate predictors, except for albumin (Supplementary Table S3). Albumin was missing for 16.7% of the patients because it is not part of standard laboratory work for emergency abdominal patients in Greece. As recommended, we imputed the missing values before model development using Multiple Imputation by Chained Equations [11, 18]. All candidate predictors and the outcome variable entered the imputation model [12], which was applied with predictive mean matching for continuous variables, logistic regression for binary variables and multinomial logistic regression for categorical variables with more than two levels. Ten imputed datasets were created from ten iterations.

### Handling of predictors

Nonlinearity was examined for each continuous predictor variable (age, albumin, BUN and WBC) by inspecting lowess-smoothed scatterplots of predictor values against the log-odds of serious complication event. Based on the graphical inspections (Supplementary Fig. S1), age was modelled with a linear term when greater than 40 years (otherwise, a zero coefficient was used), albumin was kept on its original continuous (linear) scale, BUN was modelled as linear when below 40 mg/dl, and WBC was modelled using a restricted cubic spline with knots (slopes) at quartiles. For ASA score, we combined extreme categories and used three levels (ASA I/II, ASA III, and ASA IV/V) to ensure sufficient sample size per level. All other categorical predictors were binary (yes/no) variables.

### Model-building procedures

Our approach to model development started by considering a predefined set of 11 candidate predictors in a single logistic regression model. By handling the predictors as described above, the total degrees of freedom were 14, leading to an EPV ratio of 12.5 in our development dataset. No univariate filtering or stepwise algorithm was used for selecting predictors to avoid estimation instability and bias of the model coefficients [19, 20]. To mitigate the risk of developing an overfitted model that captures local data noise, we used the Least Absolute Shrinkage and Selection Operator (LASSO) method to apply a penalty term to the model’s likelihood function and shrink the regression coefficients, such that some predictors may be entirely eliminated from the model [17]. This approach is expected to result in a parsimonious model that produces less extreme predictions on average when applied to new external datasets [17]. To address missing data, we created a stacked set of the 10 imputed datasets and set a weight to each patient equal to 0.1. In order to apply the LASSO, an optimal penalty factor (*λ*) must be determined, but this may differ across imputed datasets. Therefore, tenfold cross-validation was performed in each imputed dataset to derive ten respective *λ*’s minimizing the mean squared error between the observed and the predicted probability of a complication event. The mean *λ* was then used on the weighted stacked set to derive the LASSO model coefficients [12]. The λ values ranged from 0.018 to 0.029 (mean 0.025). In addition, post-estimation shrinkage based on optimism-corrected calibration statistics was applied to the regression coefficients as described further below. The model derived from this process was abbreviated as SEAL (SErious complications After Laparotomy).

### Model performance and validity

Model performance was assessed according to a recommended framework for evaluating clinical prediction models that proposes to examine discrimination with the c-statistic, calibration with summary statistics and graphs, overall performance with the Brier score and clinical usefulness with decision curve analysis (DCA) [21]. All performance measures were estimated with correction for optimism.

A scaled Brier score was used to quantify overall prediction accuracy, which can be interpreted as an *R*^2^-type measure representing the amount of prediction error in a null model (an uninformative model predicting the average risk) that is improved by the model under evaluation [22]. The c-statistic was used as a measure of discrimination, which equals the area under the receiver operating characteristic curve in logistic regression, and is interpreted as the probability that a randomly selected subject who experienced a complication event will have a higher predicted risk than a randomly selected subject who did not experience such an event. The c-statistic ranges from 0.5 to 1.0, with 1.0 indicating perfect discriminative ability and 0.5 indicating that the model is performing no better than random chance. For the assessment of calibration, i.e. the degree of agreement between observed and predicted risks, we calculated the calibration intercept (comparing the average predicted risk with the overall observed rate) and the calibration slope (measuring how extreme the models’ predictions are), which have ideal values of 0 and 1, respectively [23]. Additionally, we constructed lowess-smoothed curves to visually inspect the calibration. Regarding clinical utility, DCA was performed to gain insight into the range of risk thresholds to label a patient as ‘high risk for serious complication’ that would have highest net benefit (NB) for decision-making in practice. NB is the difference between the proportion of true positives (labelled as high risk pre-operatively and then going on to have a serious postoperative complication) and the proportion of false positives (labelled as high risk but not going on to have a complication) weighted by the odds of the selected threshold for the high-risk label. At any given risk threshold, the model with higher NB is the preferred model. We constructed DCA curves to visually inspect NB across a wide range of risk thresholds [24].

Correction of performance measures for optimism was achieved with Bootstrap resampling. Following multiple imputation of missing data, 200 Bootstrap samples were produced by randomly sampling with replacement from each of the 10 imputed datasets, and the results were averaged across datasets to derive optimism-corrected performance measures [25]. A detailed description of this process is presented in Supplementary methods. We then used the optimism-corrected calibration slope as a post-estimation shrinkage factor by which the SEAL coefficients were multiplied, to obtain the model’s final coefficients [12]. At this point, the model’s intercept was re-estimated using the shrunk linear predictor as offset variable to ensure model calibration was maintained. To illustrate the results, we report both apparent performance measures before applying the corrections and optimism-corrected measures.

### Model presentation

The SEAL model was presented with an equation and an example of calculating the predicted risk for a new hypothetical patient. A nomogram was constructed to enable clinicians to visually assess the importance of each predictor (by the length of the respective line) and assign points to combinations of predictors that can be easily mapped on a risk scale [19].

### Benchmarking against ACS-NSQIP

To establish a minimum benchmark for the performance of our new SEAL model, we applied the ACS-NSQIP model on the same cohort of patients. ACS-NSQIP predicted probabilities of serious complication after EL were obtained for each patient by entering all required data into the online ACS NSQIP Surgical Risk Calculator. However, previous investigations have noted, that case-mix in the HELAS cohort is significantly different from the ACS-NSQIP development cohort (for example, EL patients vs. various specialties, 30-day mortality 16.3% vs. 1.3%, respectively) [10]. To establish a best possible benchmark for the SEAL model, we recalibrated the ACS-NSQIP predictions, by adjusting its calibration intercept and slope when applied to HELAS patients using Cox’s logistic recalibration method [26]. Of note, the ACS-NSQIP model defines serious complications somewhat differently than our definition of primary outcome, by including cardiac arrest, myocardial infarction, pneumonia, progressive renal insufficiency, acute renal failure, pulmonary embolism, deep vein thrombosis, return to the operating room, deep incisional and organ-space surgical site infection, systemic sepsis, unplanned intubation, urinary tract infection and wound disruption [9]. Using this definition of serious complications, we estimated (externally validated) performance measures for the ACS-NSQIP model when applied to HELAS patients.

### Heterogeneity assessment

As this was a multicenter study, we examined SEAL model performance by accounting for clustering (intraclass correlation) by center using Internal–External Cross-Validation (IECV), so that the model was constructed in all but one centers and validated in a holdout center [27]. For this process, we used data from 7 of the 11 centers that contributed at least 50 patients to ensure stability of model estimation. Random-effects meta-analysis was then conducted to estimate overall performance measures from holdout centers and provide an assessment of heterogeneity. To investigate between-center-variation when applying the ACS-NSQIP model, we performed random-effects meta-analysis of center-specific c-statistic, calibration intercept and slope [28]. Prediction intervals were calculated for each measure to indicate expected predictive performance of the models in a new center.

### Software

All statistical analyses were performed using R version 4.2.2.

## Results

### Participants and outcome

In total, data from 633 eligible patients were analyzed. Patient mean age was 66 years (SD, 16.7 years) and 281 (43%) were classified as ASA status III/IV. The most common reason for performing EL in this cohort was gastrointestinal obstruction (39%), followed by perforation (36%) and ischemia (15%). Detailed demographics, clinical characteristics, and unadjusted associations of candidate predictors with 30-day serious complications are presented in Table 1. During the 30-day postoperative follow-up, 175 (28%) patients experienced a serious complication graded III or greater with the Clavien-Dindo classification. Of those, 50 (29%) patients returned to the operating room, while 66 (38%) patients were transferred from the ward to the ICU. Twenty-two (13%) patients had a deep incisional or an organ/space surgical site infection (SSI), 20 (11%) had a deep wound dehiscence, 93 (53%) developed postoperative sepsis, while 59 (34%) had septic shock. Median LOS was 11.7 days (IQR: 4.6–24.7) amongst complicated cases. Using the ACS-NSQIP definition, 190 (30%) patients were classified as having serious complications within 30 days of EL, whereas 131 (21%) patients experienced an event according to both definitions.

**Table 1:** Demographics, clinical characteristics and unadjusted predictor effects for 633 patients undergoing emergency laparotomy in Greece, 2020–2021

Characteristic	All patients (*n* = 633)	With serious complication (*n* = 175)	Without serious complication (*n* = 458)	Crude (unadjusted) effect	
				OR (95% CI)	*p*-value
Age (years)	66.2 ± 16.7	70.5 ± 15.5	64.5 ± 16.8	1.03 (1.01–1.04)	< 0.001
Male gender	341 (53.9)	88 (50.3)	253 (55.4)	0.82 (0.58–1.16)	0.25
BMI (kg/m^2^)	26.5 ± 5.3	26.5 ± 6.0	26.6 ± 5.1	0.99 (0.97–1.03)	0.88
Diagnosis					0.049
Obstruction	247 (39.0)	52 (29.7)	195 (42.7)	1.00	
Ischemia	94 (14.8)	30 (17.1)	64 (14.0)	2.50 (1.40–4.48)	
Perforation	226 (35.7)	67 (38.3)	159 (34.8)	1.76 (1.03–2.99)	
ASA score					< 0.001
I/II	351 (55.5)	48 (27.4)	302 (66.2)	1.00	
III	171 (27.1)	53 (30.3)	118 (25.9)	2.83 (1.81–4.41)	
IV/V	110 (17.2)	74 (42.2)	36 (7.9)	12.93 (7.83–21.35)	
Missing	1 (0.2)	1 (0.1)	0 (0)		
Functional status					
Dependent status	187 (29.6)	91 (52.3)	96 (21.1)	4.11 (2.83–5.97)	< 0.001
Missing	2 (0.3)	1 (0.6)	1 (0.2)		
Sepsis/Septic shock	106 (16.7)	67 (38.3)	39 (8.5)	6.65 (4.25–10.4)	< 0.001
Disseminated Cancer	73 (11.6)	24 (13.7)	49 (10.7)	1.32 (0.78–2.23)	0.29
Missing	1 (0.2)	0 (0)	1 (0.2)		
Preoperative dyspnoea	55 (8.8)	24 (14.0)	31 (6.8)	2.22 (1.26–3.91)	0.005
Missing	8 (12.6)	5 (2.8)	3 (0.6)		
Albumin (g/dl)	3.4 ± 0.9	2.9 ± 0.6	3.5 ± 0.9	0.28 (0.20–0.40)	< 0.001
Missing	106 (16.7)	31 (17.7)	75 (16.4)		
Preoperative steroids	55 (8.8)	25 (14.5)	30 (6.6)	2.40 (1.37–4.21)	0.002
Missing	7 (1.1)	4 (2.3)	3 (0.6)		
Ascites	81 (12.8)	37 (21.1)	44 (9.6)	2.51 (1.56–4.05)	< 0.001
Missing	1 (0.2)	0 (0)	1 (0.2)		
BUN (mg/dl)	25.3 ± 16.7	30.4 ± 18.8	23.4 ± 15.4	1.05 (1.03–1.07)	< 0.001
WBC (× 10^^9^ / l)	12.5 ± 7.7	13.9 ± 10.7	12.0 ± 6.2		0.001
RCS term 1				0.94 (0.87–1.01)	
RCS term 2				1.11 (1.02–1.20)	

Continuous variables are reported as mean ± SD, categorical variables as *n* (%)

*OR* odds ratio, *CI* confidence Interval, *BMI* Body Mass Index, *ASA* American Society of Anesthesiologists; BUN: Blood Urea Nitrogen; WBC: White Blood Cells; RCS: Restricted Cubic Spline

### The SEAL model

**Fig. 1:**
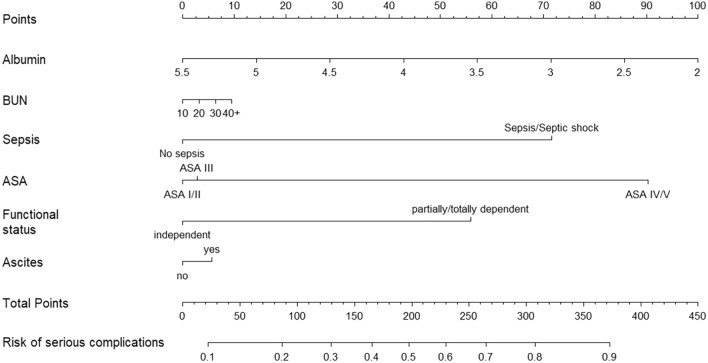
Prediction nomogram of the SEAL model for the risk of 30-day serious postoperative complications. *BUN* blood urea nitrogen, *ASA* American Society of Anaesthesiologists. Albumin is expressed in g/dl, BUN in mg/dl

Following LASSO estimation, 6 of the 11 candidate predictors of serious complications were kept in the final logistic regression model, namely, preoperative albumin, BUN, presence of sepsis or septic shock, ASA class, dependent functional status and presence of ascites. The final model coefficients (after post-estimation shrinkage was applied) are shown in Table 2, together with the full model equation and an example of calculating a predicted risk for a new hypothetical EL patient. The model’s nomogram is illustrated in Fig. 1.

**Table 2:** SEAL model regression coefficients, equation and indicative example of calculating the risk of serious complication for new patient who will undergo emergency laparotomy

SEAL model coefficients		SEAL model equation	Example
Intercept	– 0.5331	Linear predictor	Hypothetical EL patient: Albumin: 2.6 BUN: 30 Presents with sepsis ASA III Independent functional status No ascites
Albumin	– 0.3193	LP = – 0.5331–0.3193 * albumin + 0.0039 (if BUN ≥ 40) + 0.8956 (if Sepsis or Septic shock) + 0.0418 (if ASA III) + 1.1492 (if ASA IV/V) + 0.7048 (if dependent functional status) + 0.0832 (if ascites)	
BUN	0.0039		
Sepsis or septic shock	0.8956		
ASA III	0.0418	Probability of 30-day serious complication $=\frac{1}{1+{e}^{-LP}}$	Linear predictor $= - 0.5331 - 0.3193\times 2.6 + 0.0039\times 0 + 0.8956\times 1 + 0.0418\times 1 + 1.1492\times 0 + 0.7048\times 0 + 0.0832\times 0 =\boldsymbol{ }- 0.42588$
ASA IV or V	1.1492		
Dependent functional status	0.7048		Predicted risk of serious complication $= \frac{1}{{1 + e^{0.42588} }} = 0.395 \, {\text{or}} \, 39.5\%$
Ascites	0.0832		From the nomogram, this patient would get about 165 points, corresponding to a risk of about 0.4 or 40%

*BUN* blood urea nitrogen, *ASA* American Society of Anesthesiologists, *EL* emergency laparotomy

### Predictive performance

**Fig. 2:**
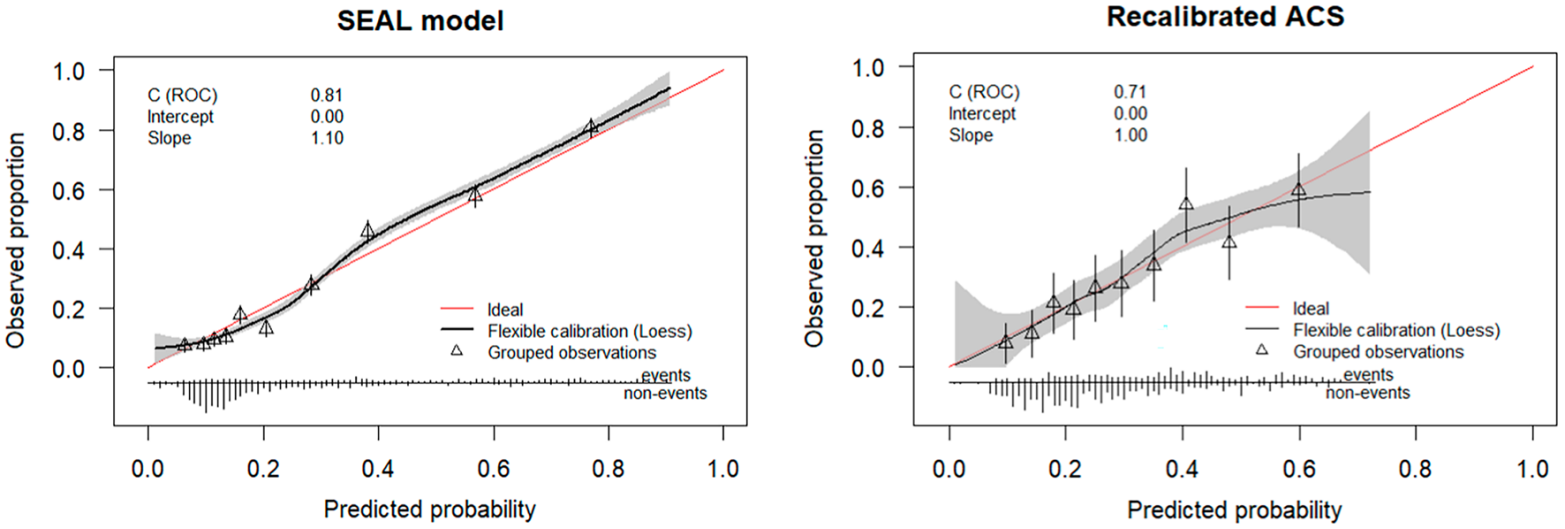
Calibration curves of the SEAL and recalibrated ACS-NSQIP models for serious postoperative complications. A loess line (black) depicts the degree of agreement between predicted probabilities and observed proportions, while the red line represents the ideal calibration. *ROC* Receiver Operating Characteristic area, *Loess* locally estimated scatterplot smoothing

**Fig. 3:**
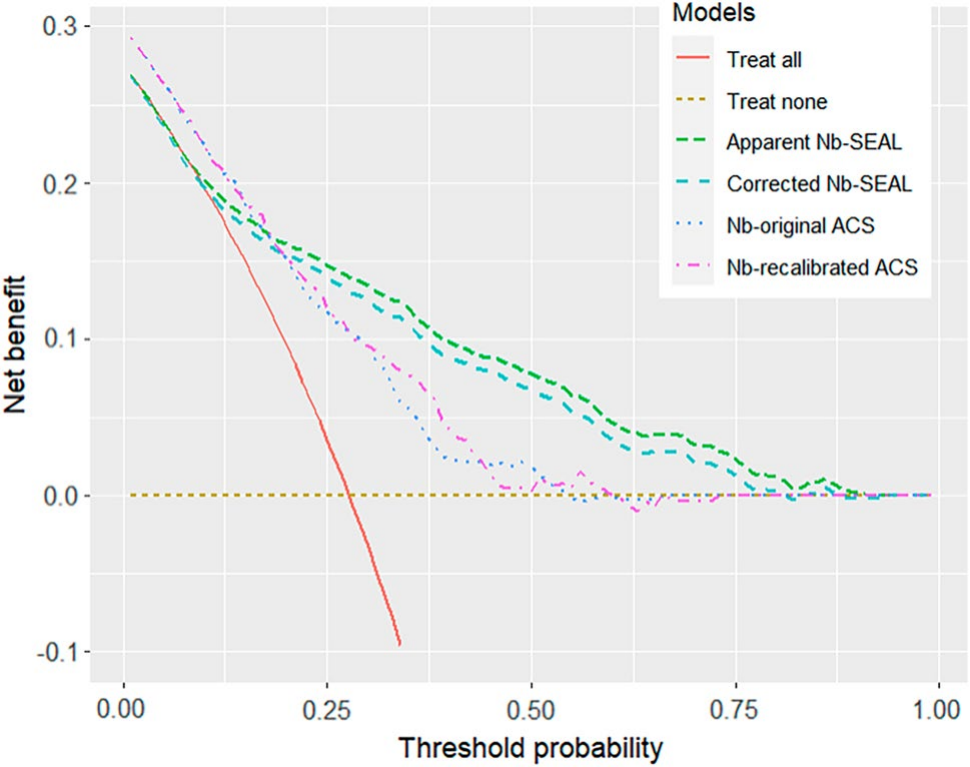
Decision Curve Analysis of the SEAL and ACS-NSQIP models for serious postoperative complications. The Net Benefit is plotted against risk thresholds for naming a patient as high risk for serious complications. *Nb* Net Benefit

Measures of the predictive performance for SEAL, contrasted against those of the ACS-NSQIP model, are presented in Table 3. SEAL had better predictive performance as indicated by a higher optimism-corrected scaled Brier score (25.1%, 95% CI 24.1–26.1%), compared to the scaled Brier score of the recalibrated ΑCS-NSQIP model (10.8%, 95% CI 9.2–12.2%) when applied to the HELAS cohort. Discrimination was good for both models, with the SEAL model retaining a high c-statistic value after internal validation (0.80, 95% CI 0.79–0.81). ROC curves for both models can be seen in Supplementary Fig. S2. Calibration of the SEAL and recalibrated ACS-NSQIP model can be inspected graphically in Fig. 2, while the calibration curve of the unadjusted ACS-NSQIP is seen in Supplementary Fig. S3. Before updating, the ACS-NSQIP model systematically underestimated the risk of serious complications in our patient cohort (calibration intercept: 0.27, 95% CI 0.21–0.33), while the SEAL model was closer to the ideal diagonal line of calibration. As seen in Table 3, the LASSO method led to over-shrinkage of the regression coefficients (calibration slope: 1.10, 95% CI 1.08–1.13), leading to predictions, that would be less extreme, than what they should be, which was mediated by internal validation (corrected slope: 1.01, 95% CI 0.99–1.03). From the results of DCA in Fig. 3, the SEAL model had higher NB across a wider range of risk thresholds compared to the recalibrated ACS-NSQIP model, retaining superiority after correction for optimism.

**Table 3:** Predictive performance statistics of the newly developed SEAL model (apparent and optimism-corrected measures) and the ACS-NSQIP model (external validation) when applied to the HELAS cohort to predict the risk of serious 30-day postoperative complications

Performance measure	SEAL model			ACS-NSQIP model	
	Apparent (95% CI)^a^	Mean optimism	Optimism-corrected (95% CI)	Unadjusted (95% CI)	Recalibrated (95% CI)
C-statistic	0.81 (0.80,0.83)	0.01	0.80 (0.79, 0.81)	0.71 (0.69,0.72)	0.71 (0.69,0.72)
Intercept	0.00 (-0.06,0.06)	0.00	0.00 (-0.01, 0.01)	0.27 (0.21,0.33)	0.00 (0.00,0.00)
Slope	1.10 (1.08,1.13)	0.09	1.01 (0.99, 1.03)	1.03 (0.97,1.11)	1.00 (0.98,1.03)
Brier scaled (%)	28.1 (27.6,29.0)	3.0	25.1 (24.1,26.1)	9.3 (7.9,10.4)	10.8 (9.2,12.2)

*CI* confidence interval, *HELAS* Hellenic Emergency Laparotomy Study, *SEAL* SErious complications After Laparotomy, *ACS-NSQIP* American College of Surgeons National Surgical Quality Improvement Program

^a^Apparent predictive performance implies assessment directly in the derivation cohort that yields an optimistic estimate of model performance, because the regression coefficients are optimized for the derivation cohort. The degree of optimism was estimated using Bootstrap resampling and the estimated mean optimism was subtracted from the apparent performance to indicate the expected model performance for future patients similar to the derivation cohort

### Heterogeneity assessment

Results of the IECV analysis indicated that the SEAL model is expected to have adequate discriminative ability in new centers or settings (95% prediction interval for c-statistic 0.64–0.93), but heterogeneity was more notable for the calibration measures (Supplementary Figs. S4–S6), implying a possible need for model recalibration in new settings. By contrast, the random-effects meta-analysis of hospital-specific performance measures for the ACS-NSQIP model showed substantial heterogeneity across centres with wide prediction intervals (Supplementary Figs. S7–S9).

## Discussion

This study developed and internally validated a new prognostic model for serious complications after EL. The derived SEAL model is based on six preoperative predictor variables, comprising readily available biomarkers (albumin and BUN) and indices of patient status at presentation (ASA score, dependent functional status, presence of ascites and organ dysfunction as indicated by sepsis or septic shock). State-of-the-art statistical methods were employed to overcome common barriers when developing SEAL, duly addressing the issues of overfitting, optimism, missing data and heterogeneity [11, 29]. The results demonstrated that SEAL performed well in predicting serious complications, defined as Clavien-Dindo grade ≥ 3, in a diverse multicentre cohort of EL patients in Greece. Based on multiple measures of predictive performance, SEAL not only outperformed the minimum threshold of a “null model” that predicts the average risk (28% in this cohort), but compared favourably when benchmarked against the rival ACS-NSQIP model for all predictive performance metrics, including DCA.

As evident from SEAL’s nomogram, serum albumin constitutes an important predictor of serious postoperative complications in EL patients. Low serum albumin level has been repeatedly and strongly associated with mortality after EL in several previous studies [13]. In a recent study of the association between albumin and wound-related complications in EL, hypoalbuminemia (serum albumin < 3.5 g/dl) correlated with increased risks of surgical site infections, wound dehiscence and increased LOS [30]. In the emergency setting, albumin has been used as a predictor of perioperative mortality for the calculation of the Emergency Acuity Surgery Score [31], and has been incorporated as a predictor of several other outcomes in the POTTER model [8]. Moreover, albumin is an accepted biomarker for malnutrition and the ongoing MATS trial is currently investigating its predictive importance in general surgery patients (NCT05393752). BUN was another biomarker that was included in the SEAL model, which agrees with previous studies that reported preoperative urea levels to be significantly elevated in non-survivors of emergency abdominal surgery in the UK [32], and BUN > 40 mg/dl to be independently associated with mortality in the ACS-NSQIP cohort of EL patients in the USA [33].

Another important predictor in SEAL was the dependent preoperative functional status. A recent study of more than 1,000 EL patients found that higher Eastern Cooperative Oncology Group (ECOG) performance scores were associated with 30-day mortality, recognising the prognostic importance of measures of frailty, due to the specific perioperative care that frail individuals may warrant [34]. Presence of ascites also contributed to the prediction of serious complications after EL in this study, which is line with previous reports of high correlations between ascites and poor outcomes in emergency general surgery, including abdominal wall dehiscence and pulmonary complications [35]. Chronic liver disease complicates the postoperative course, often necessitating critical care admission for fluid and electrolyte management [35]. Ascites is also present in patients with disseminated malignancies, who comprise a special subset of EL patients often requiring palliative surgery for gastrointestinal obstruction or perforation. Malignant ascites was an independent predictor of postoperative death in both the obstruction and perforation subgroups in a cohort of disseminated cancer patients undergoing emergency surgery in the USA [14].

Important methodological issues related to risk of bias and applicability of clinical prediction models, as emphasized in the recently published Prediction model Risk Of Bias ASsessment Tool (PROBAST) [18], were attentively considered and addressed in this study. Our approach to model development comprised carefully selecting candidate predictors by literature review, modelling nonlinearities in continuous predictor variables, handling missing values with multiple imputation, applying LASSO shrinkage to ensure that predictions for future patients would not be too extreme, and using Bootstrap resampling to obtain optimism-corrected estimates of future predictive performance. In addition, we exploited the critical opportunity offered by the multicentre nature of the study to investigate heterogeneous effects in predictive associations. Heterogeneity is particularly relevant as predictive performance and generalizability of a developed model may be limited by between-center variability in the distribution of predictor values, the predictor-outcome associations and/or baseline risks or outcome incidence [27]. To inspect whether heterogeneity would actually be a concern when SEAL would be implemented in clinical practice, we adopted a meta-analytic framework and applied the IECV method [27, 36]. Our results implied that SEAL’s performance is expected to be robust in terms of discrimination, but more uncertain regarding calibration in new centers or settings. It is therefore likely that SEAL may require local revisions prior to implementation in some settings, which may involve simple intercept update, recalibration or rescaling of regression coefficients or even full re-estimation of all the regression coefficients [37]. This might prove a limitation for implementing SEAL in other settings and populations; nevertheless, SEAL performed better than ACS-NSQIP in this respect.

We argue in our approach that examining the performance of SEAL in isolation makes little sense and much more insight on its potential utility can be gained from benchmarking against rival models. To our knowledge, there is limited research on predictive analytics for EL complications and the only rival model for SEAL is the ACS-NSQIP, which is largely endorsed by the surgeons' community to drive surgical decision making and informed consent. In a previous investigation, we showed that ACS-NSQIP produced accurate predictions of postoperative mortality in the HELAS cohort and outperformed three other commonly cited prognostic models [38]. Despite our endorsement, we found that using ACS-NSQIP in practice is burdensome and requires entering data for a large number of 21 preoperative factors in an external online calculator, which is proprietary with undisclosed equation. We therefore sought a simpler, more transparent and applicable model for our setting that would perform at least equally well as the ACS-NSQIP in predicting post EL complications. To make the comparison as meaningful and fair as possible, we benchmarked the optimism-corrected SEAL performance metrics against the recalibrated ACS-NSQIP performance metrics. The former ensured that unbiased non-optimistic estimates of future prediction performance were obtained for SEAL, whereas the latter provided most accurate predictions from ACS-NSQIP when applied to the external HELAS cohort. Additionally, we performed DCA to investigate the problem in terms of the threshold probability above which a decision maker would deem the expected value of intervention to be greater than not doing so [24]. Our results indicated that utilising SEAL as a general prognostic model to inform personal decisions should be expected to have higher NB compared to ACS-NSQIP when risk thresholds for defining a “high-risk” patient exceed 20%. It is understood that EL patients deemed high-risk for serious complications by the SEAL model would then be managed within an appropriate care bundle, that ensures intensive care and advanced complication management options (e.g. endoscopy and interventional radiology) are available for those patients.

The following limitations need to be considered. First, despite our EPV ratio of 12.5 generally regarded sufficient for model development, recent simulation studies suggested EPV ratios exceeding 20 as desirable to prevent overfitting [18, 39]. Nevertheless, the application of penalized regression methods is expected to have largely mitigated overfitting during the development of SEAL. Second, the prospective design of the study ensured that clear and objective criteria for data collection were applied in all participating centers, but predictors such as the ASA score are always prone to subjective assessment by attending physicians and this might create higher-than-anticipated heterogeneity in other settings. Third, participating centres were tertiary-care hospitals and our derivation cohort might not be a true population-based or nationally representative sample of EL patients in Greece; thereby, performing external validation studies of SEAL in broader settings is crucial [40]. Both temporal validation in the same hospitals and broader external validation in other settings with different case-mix are required to confirm SEAL’s reproducibility and transportability, respectively. Finally, we should acknowledge that the benchmarking of SEAL against ACS-NSQIP was merely indirect because the definition of outcome is not exactly identical for the two models.

## Conclusion

The newly developed SEAL model is a simple and promising model for accurate individualized predictions of the risk of serious complications after EL. Future external validation studies should confirm SEAL’s reproducibility in similar patient populations and appraise its transportability across diverse settings.

### Supplementary Information

Below is the link to the electronic supplementary material.

Supplementary file1 (DOCX 382 KB)

## Data Availability

The data supporting this study’s findings are available upon reasonable request.
