# Supplementary material for: Development and internal validation of a clinical prediction model for serious complications after emergency laparotomy
Source: Eur J Trauma Emerg Surg. 2023 Aug 31;50(1):283–93. doi: 10.1007/s00068-023-02351-4 (PMC10923974; doi:10.1007/s00068-023-02351-4)
Supplement: Supplementary file 1 — Supplementary file1 (DOCX 382 KB) [file 68_2023_2351_MOESM1_ESM.docx]

**Supplementary material**

**Development and internal validation of a clinical prediction model for serious complications after emergency laparotomy**

***European Journal of Trauma and Emergency Surgery***

**Authors**

Stamatios Kokkinakis^1^ (ORCID: 0000-0003-2733-314X), Evangelos I. Kritsotakis^2^ (ORCID: 0000-0002-9526-3852), Konstantinos Paterakis^1^, Garyfallia-Apostolia Karali^1^, Vironas Malikides^1^, Anna Kyprianou^1^, Melina Papalexandraki^1^, Charalampos S. Anastasiadis^3^ Odysseas Zoras^3^, Nikolas Drakos^4^, Ioannis Kehagias^4^, Dimitrios Kehagias^4^, Nikolaos Gouvas^5^, Georgios Kokkinos^5^, Ioanna Pozotou^5^, Panagiotis Papatheodorou^5^, Kyriakos Frantzeskou^5^, Dimitrios Schizas^6^, Athanasios Syllaios^6^, Ifaistion M Palios^7^, Konstantinos Nastos^8^, Markos Perdikaris^8^, Nikolaos V Michalopoulos^8^, Ioannis Margaris^8^, Evangelos Lolis^9^, Georgia Dimopoulou^9^, Dimitrios Panagiotou^10^, Vasiliki Nikolaou^10^, Georgios K. Glantzounis^11^, George Pappas-Gogos^11^, Kostas Tepelenis^11^, Georgios Zacharioudakis^12^, Savvas Tsaramanidis^12^, Ioannis Patsarikas^12^, Georgios Stylianidis^13^, Georgios Giannos^13^, Michail Karanikas^14^, Konstantinia Kofina^14^, Markos Markou^14^, Emmanuel Chrysos^1^, Konstantinos Lasithiotakis^1^ (ORCID: 0000-0002-6538-0951)

**Affiliations**

1. Department of General Surgery, University Hospital of Heraklion, University of Crete, School of Medicine, Greece.

2. Laboratory of Biostatistics, School of Medicine, University of Crete, Heraklion, Crete, Greece.

3. Department of Surgical Oncology, University Hospital of Heraklion, University of Crete, School of Medicine, Greece.

4. Department of Surgery, University General Hospital of Patras, School of Medicine, University of Patras, Patras, Greece.

5. Department of Surgery, General Hospital of Nicosia, School of Medicine, University of Cyprus, Nicosia, Cyprus.

6. First Department of Surgery, National and Kapodistrian University of Athens, Laikon General Hospital, Athens, Greece.

7. Second Propaedeutic Department of Surgery, National and Kapodistrian University of Athens, Laikon General Hospital, Athens, Greece.

8. Department of Surgery, University General Hospital Attikon, School of Medicine, University of Athens, Athens, Greece.

9. Department of Surgery, General Hospital of Volos, Volos, Greece.

10. Department of Surgery, General Hospital of Trikala, Trikala, Greece.

11. Department of Surgery, University Hospital of Ioannina, Greece.

12. Department of Surgery, Ippokrateion General Hospital of Thessaloniki, School of Medicine, Aristotle University of Thessaloniki, Thessaloniki, Greece.

13. Second Department of Surgery, Evangelismos General Hospital, Athens, Greece.

14. Department of Surgery, University General Hospital of Alexandroupolis, School of Medicine, University of Thrace, Alexandroupolis, Greece.

**Correspondence:** Evangelos I. Kritsotakis, Laboratory of Biostatistics, School of Medicine, University of Crete, 71003, Heraklion, Crete, Greece, Email: e.kritsotakis@uoc.gr

| Contents | Page |
| --- | --- |
| Table S1. Inclusion and Exclusion criteria | 4 |
| Table S2. TRIPOD checklist | 5-6 |
| Table S3. Missing values for important variables | 7 |
| Figure S1. Relationship of continuous predictors with log-odds of outcome | 8 |
| Supplementary methods. Combination of internal validation with multiple imputation | 9 |
| Figure S2. ROC curves for the SEAL and ACS-NSQIP models | 10 |
| Figure S3. Calibration curve of the unadjusted ACS-NSQIP | 11 |
| Figure S4. Internal-External Cross-Validation of c-statistic estimates for the SEAL model | 12 |
| Figure S5. Internal-External Cross-Validation of slope estimates for the SEAL model | 13 |
| Figure S6. Internal-External Cross-Validation of intercept estimates for the SEAL model | 14 |
| Figure S7. Meta-analysis of hospital-specific c-statistic estimates for the ACS-NSQIP model | 15 |
| Figure S8. Meta-analysis of hospital-specific slope estimates for the ACS-NSQIP model | 16 |
| Figure S9. Meta-analysis of hospital-specific intercept estimates for the ACS-NSQIP model | 17 |
| Table S1: Inclusion and Exclusion criteria   \| **Inclusion criteria:** \| \| --- \| \| - Age >18yrs - Emergency laparotomy (operation simultaneously with resuscitation usually within one hour) or - urgent (operation as soon as possible after resuscitation, within 24hrs) - Operation in the gastrointestinal tract: - Open or laparoscopic, or laparoscopically assisted procedures. - Procedures involving the stomach, small or large bowel, or rectum for conditions such as - perforation, ischaemia, abdominal abscess, bleeding or obstruction - Wash out/evacuation of intraperitoneal abscess or haematoma - Bowel resection/repair due to incarcerated/incisional hernias - Bowel resection or repair due to incarcerated umbilical, inguinal or femoral hernias - Open or laparoscopic adhesiolysis - Laparotomy/laparoscopy with inoperable pathology - Return to theatre for repair of a substantial dehiscence of major abdominal wound (i.e.“burst abdomen”) - Return to theatre after any operation (including vascular, gynaecology, urology, cardiac) meeting the criteria above - In the case of multiple procedures in the abdominopelvic cavity the patient is included if the main procedure is a general surgical one (i.e. if bowel resection happens during an open aneurysm repair it should not be included) - Any intra-abdominal procedure not identifiable within exclusion criteria should be included. \| \| **Exclusion criteria:** \| \| - Patients under 18 - Elective operation - Diagnostic laparoscopy or laparotomy where no other procedure is performed (NB, if no procedure is performed due to inoperable pathology, then include) - Appendicectomy with or without drainage of localized abscess - Cholecystectomy with or without drainage of localized abscess - Hernia repair without bowel resection - Minor abdominal wound revision - Vascular surgery - Gynaecological surgery – c-section – ruptured ectopic pregnancy - Surgery relating to organ transplantation \|   Table S2: Checklist for Transparent Reporting of a Multivariable Prediction Model for Individual Prognosis or Diagnosis (TRIPOD) |  |
|  |  |
| \| **Section/Topic** \|  \|  \| **Checklist Item** \| **Manuscript Section** \| \| --- \| --- \| --- \| --- \| --- \| \| **Title and abstract** \| \| \| \| \| \| Title \| 1 \| D;V \| Identify the study as developing and/or validating a multivariable prediction model, the target population, and the outcome to be predicted. \| Title \| \| Abstract \| 2 \| D;V \| Provide a summary of objectives, study design, setting, participants, sample size, predictors, outcome, statistical analysis, results, and conclusions. \| Abstract \| \| **Introduction** \| \| \| \| \| \| Background and objectives \| 3a \| D;V \| Explain the medical context (including whether diagnostic or prognostic) and rationale for developing or validating the multivariable prediction model, including references to existing models. \| Introduction:  1st and 2nd paragraphs \| \| 3b \| D;V \| Specify the objectives, including whether the study describes the development or validation of the model or both. \| Introduction: last paragraph \| \| **Methods** \| \| \| \| \| \| Source of data \| 4a \| D;V \| Describe the study design or source of data (e.g., randomized trial, cohort, or registry data), separately for the development and validation data sets, if applicable. \| Methods: Data source \| \| 4b \| D;V \| Specify the key study dates, including start of accrual; end of accrual; and, if applicable, end of follow-up. \| Methods: Data source \| \| Participants \| 5a \| D;V \| Specify key elements of the study setting (e.g., primary care, secondary care, general population) including number and location of centres. \| Methods: Data source \| \| 5b \| D;V \| Describe eligibility criteria for participants. \| Table S1 \| \| 5c \| D;V \| Give details of treatments received, if relevant. \| Methods: Data source \| \| Outcome \| 6a \| D;V \| Clearly define the outcome that is predicted by the prediction model, including how and when assessed. \| Methods: Outcome \| \| 6b \| D;V \| Report any actions to blind assessment of the outcome to be predicted. \| Not applicable \| \| Predictors \| 7a \| D;V \| Clearly define all predictors used in developing or validating the multivariable prediction model, including how and when they were measured. \| Methods: Predictors \| \| 7b \| D;V \| Report any actions to blind assessment of predictors for the outcome and other predictors. \| Not applicable \| \| Sample size \| 8 \| D;V \| Explain how the study size was arrived at. \| Methods: Sample size \| \| Missing data \| 9 \| D;V \| Describe how missing data were handled (e.g., complete-case analysis, single imputation, multiple imputation) with details of any imputation method. \| Methods: Missing data / Table S3 \| \| Statistical analysis methods \| 10a \| D \| Describe how predictors were handled in the analyses. \| Methods: Handling of predictors / Figure S1 \| \| 10b \| D \| Specify type of model, all model-building procedures (including any predictor selection), and method for internal validation. \| Methods: Model-building procedures / Model performance and validity / Table S4 \| \| 10c \| V \| For validation, describe how the predictions were calculated. \| Methods: Model performance and validity / Table S4 \| \| 10d \| D;V \| Specify all measures used to assess model performance and, if relevant, to compare multiple models. \| Methods: Model performance and validity / Benchmarking / Heterogeneity assessment \| \| 10e \| V \| Describe any model updating (e.g., recalibration) arising from the validation, if done. \| Methods: Benchmarking \| \| Risk groups \| 11 \| D;V \| Provide details on how risk groups were created, if done. \| Not applicable \| \| Development vs. validation \| 12 \| V \| For validation, identify any differences from the development data in setting, eligibility criteria, outcome, and predictors. \| Methods: Benchmarking \| \| **Results** \| \| \| \| \| \| Participants \| 13a \| D;V \| Describe the flow of participants through the study, including the number of participants with and without the outcome and, if applicable, a summary of the follow-up time. A diagram may be helpful. \| Results: Participants and Outcome \| \| 13b \| D;V \| Describe the characteristics of the participants (basic demographics, clinical features, available predictors), including the number of participants with missing data for predictors and outcome. \| Results: Participants and Outcome / Table 1 \| \| 13c \| V \| For validation, show a comparison with the development data of the distribution of important variables (demographics, predictors and outcome). \| NA \| \| Model development \| 14a \| D \| Specify the number of participants and outcome events in each analysis. \| Results: Participants and Outcome / Table 1 \| \| 14b \| D \| If done, report the unadjusted association between each candidate predictor and outcome. \| Table 1 \| \| Model specification \| 15a \| D \| Present the full prediction model to allow predictions for individuals (i.e., all regression coefficients, and model intercept or baseline survival at a given time point). \| Table 2, Figure 1 \| \| 15b \| D \| Explain how to the use the prediction model. \| Results: The SEAL model /Table 2 / Figure 1 \| \| Model performance \| 16 \| D;V \| Report performance measures (with CIs) for the prediction model. \| Results: Predictive performance / Table 3 / Figure 2 \| \| Model-updating \| 17 \| V \| If done, report the results from any model updating (i.e., model specification, model performance). \| (for the ACS-NSQIP model)  Results: Predictive performance / Table 3 / Figure 2-3 \| \| **Discussion** \| \| \| \| \| \| Limitations \| 18 \| D;V \| Discuss any limitations of the study (such as nonrepresentative sample, few events per predictor, missing data). \| Page 11 \| \| Interpretation \| 19a \| V \| For validation, discuss the results with reference to performance in the development data, and any other validation data. \| Pages 9-11 \| \| 19b \| D;V \| Give an overall interpretation of the results, considering objectives, limitations, results from similar studies, and other relevant evidence. \| Pages 9-11 \| \| Implications \| 20 \| D;V \| Discuss the potential clinical use of the model and implications for future research. \| Page 11 \| \| **Other information** \| \| \| \| \| \| Supplementary information \| 21 \| D;V \| Provide information about the availability of supplementary resources, such as study protocol, Web calculator, and data sets. \| Page 12 \| \| Funding \| 22 \| D;V \| Give the source of funding and the role of the funders for the present study. \| Page 12 \| |  |
|  |  |

**Table S3**: Missing values for important variables

| Variable | Missing values (%) |
| --- | --- |
| Age | 0 (0) |
| Albumin | 106 (16.7) |
| WBC | 0 (0) |
| BUN | 0 (0) |
| Disseminated cancer | 1 (0.2) |
| Respiratory status | 8 (1.3) |
| Sepsis/Septic shock | 0 (0) |
| ASA score | 1 (0.2) |
| Functional status | 2 (0.3) |
| Steroid use | 7 (1.1) |
| Ascites | 1 (0.2) |
| Serious complications | 1 (0.2) |
| ACS predictions | 8 (1.3) |
| ACS outcome | 0 (0) |

**Figure S1**. Relationship of continuous predictors with log-odds of the outcome


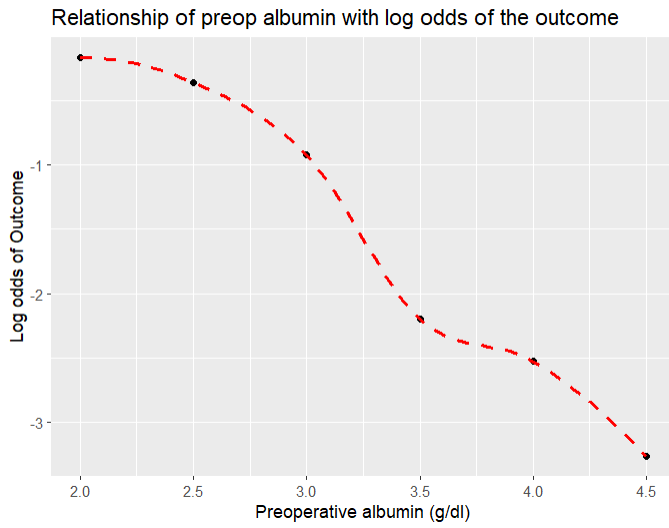

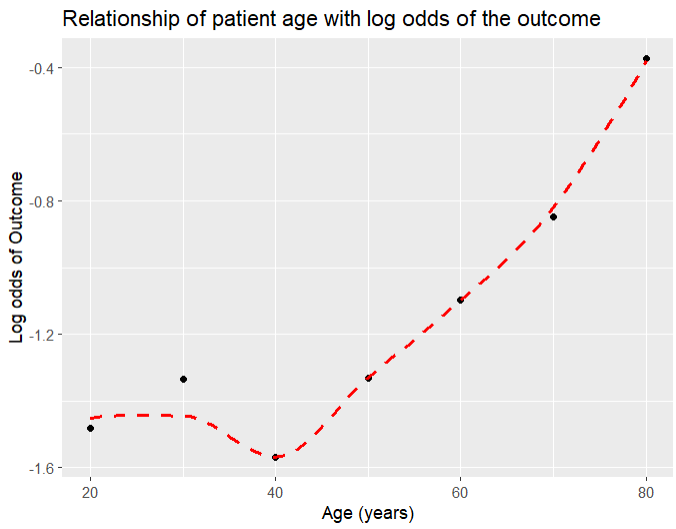


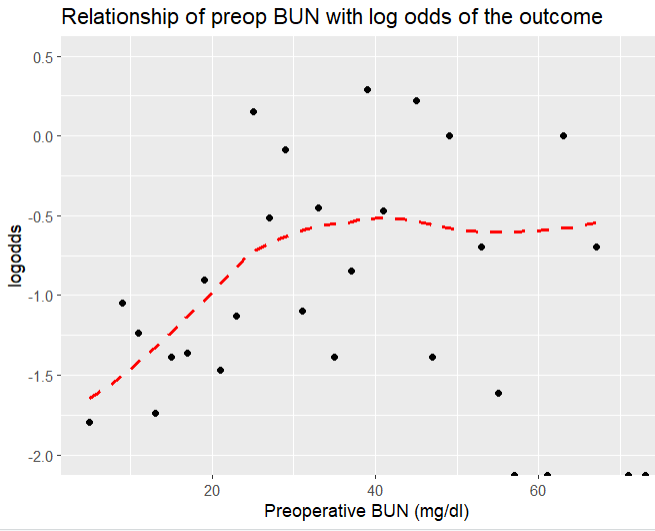

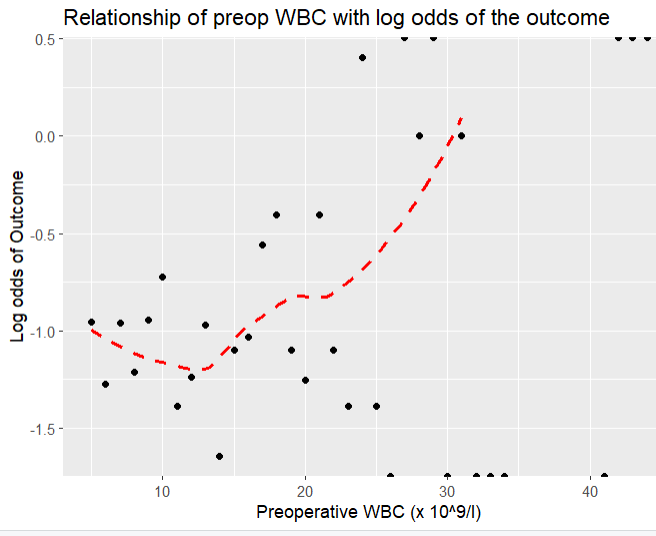


**Supplementary methods**. Internal validation with multiple imputation

The following procedure was performed for the SEAL model:

1. Within each imputed dataset, the model was fitted and its *Apparent* performance measures were calculated.
2. A bootstrap sample was drawn with replacement.
3. The whole modelling procedure was repeated in the bootstrap sample, and model performance measures in that sample were calculated (*Train*).
4. The model developed in the bootstrap sample was fitted to the original imputed dataset, and its performance measures were calculated (*Test*).
5. Optimism within the bootstrap sample is the difference: *Train-Test*
6. Previous steps were repeated 200 times and a *mean* *Optimism* was obtained.
7. The *Corrected performance* within the imputed dataset is the difference between the *Apparent* performance measure and the *mean Optimism*.
8. The mean *Corrected performance* across 10 imputed datasets was obtained for each performance measure.


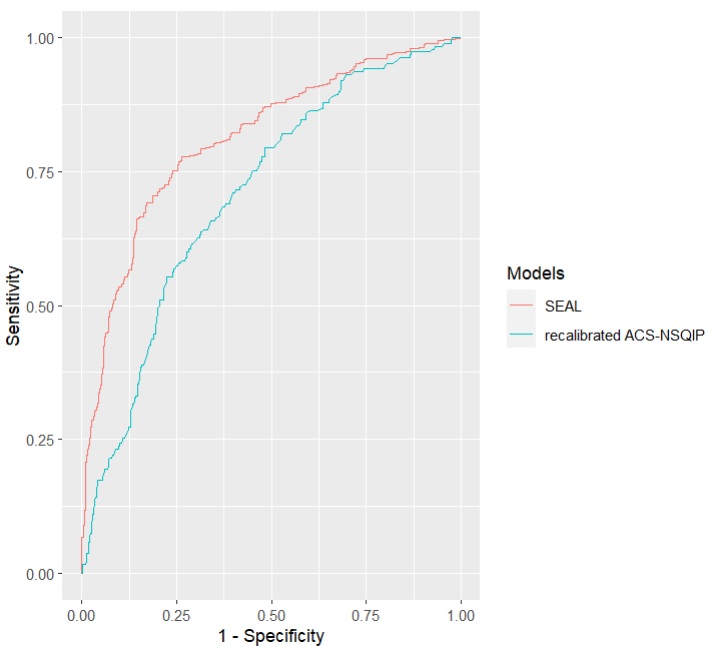
**Figure S2**.ROC curves for the SEAL and recalibrated ACS-NSQIP model

**Figure S3**. Calibration curve of the unadjusted ACS-NSQIP model for serious postoperative complications applied to the HELAS patient cohort. A loess line (black) depicts the degree of agreement between predicted probabilities and observed proportions, while the red line represents the ideal calibration. ROC: Receiver Operating Characteristic area; Loess: locally estimated scatterplot smoothing.


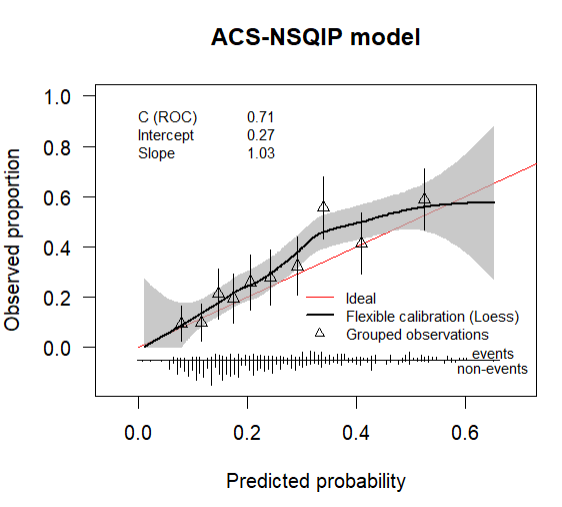


**Figure S4**. Internal-External Cross-Validation of c-statistic estimates for the SEAL model


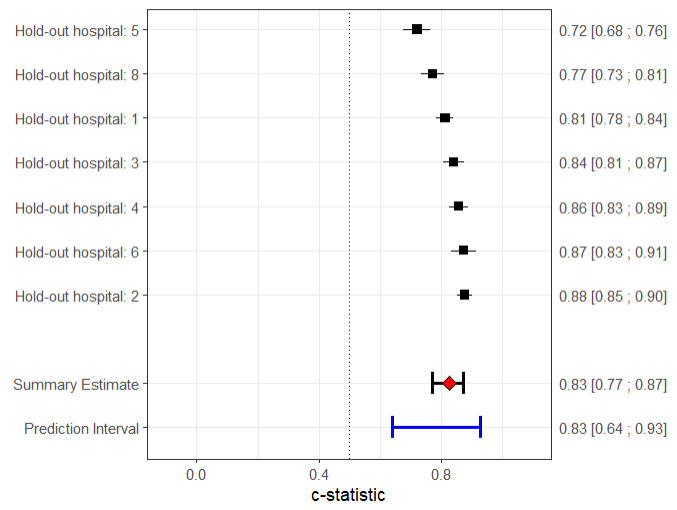


Note:

1.Meta-analysis was performed with the use of logit transformed c-statistics.

2.The prediction interval indicates a possible range of the c-statistic value in a new center.

**Figure S5**. Internal-External Cross-Validation of slope estimates for the SEAL model


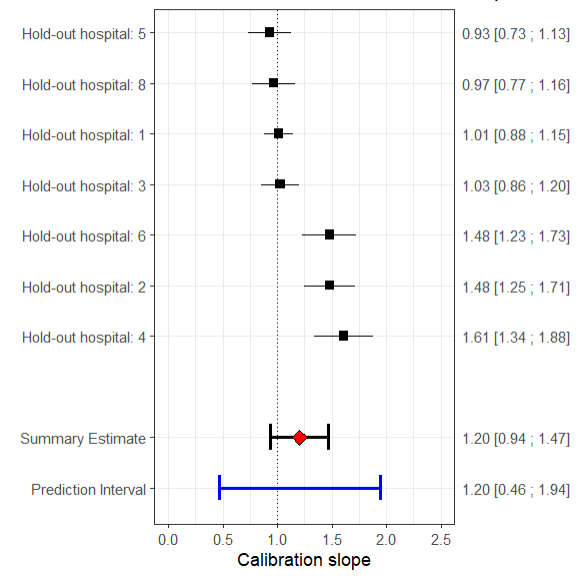


Note:

1.Meta-analysis was performed with the use of the original scale of the slopes.

2.The prediction interval indicates a possible range of the slope value in a new center.

**Figure S6**. Internal-External Cross-Validation of intercept estimates for the SEAL model


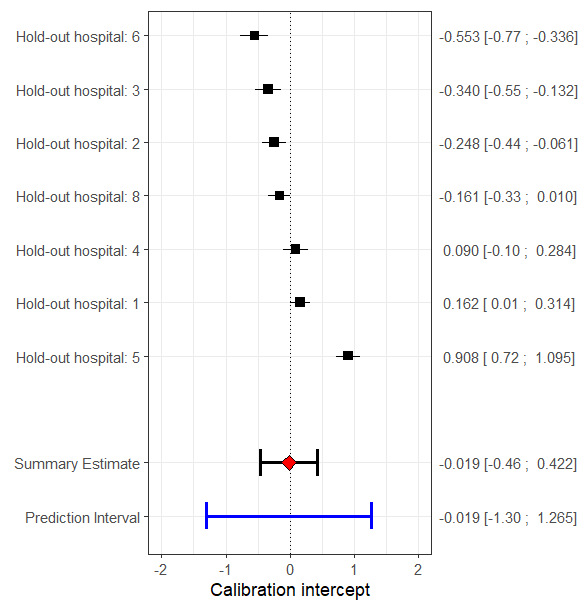


Note:

1.Meta-analysis was performed with the use of the original scale of the intercepts.

2.The prediction interval indicates a possible range of the intercept value in a new center.

**Figure S7**. Meta-analysis of hospital-specific c-statistic estimates for the ACS-NSQIP model


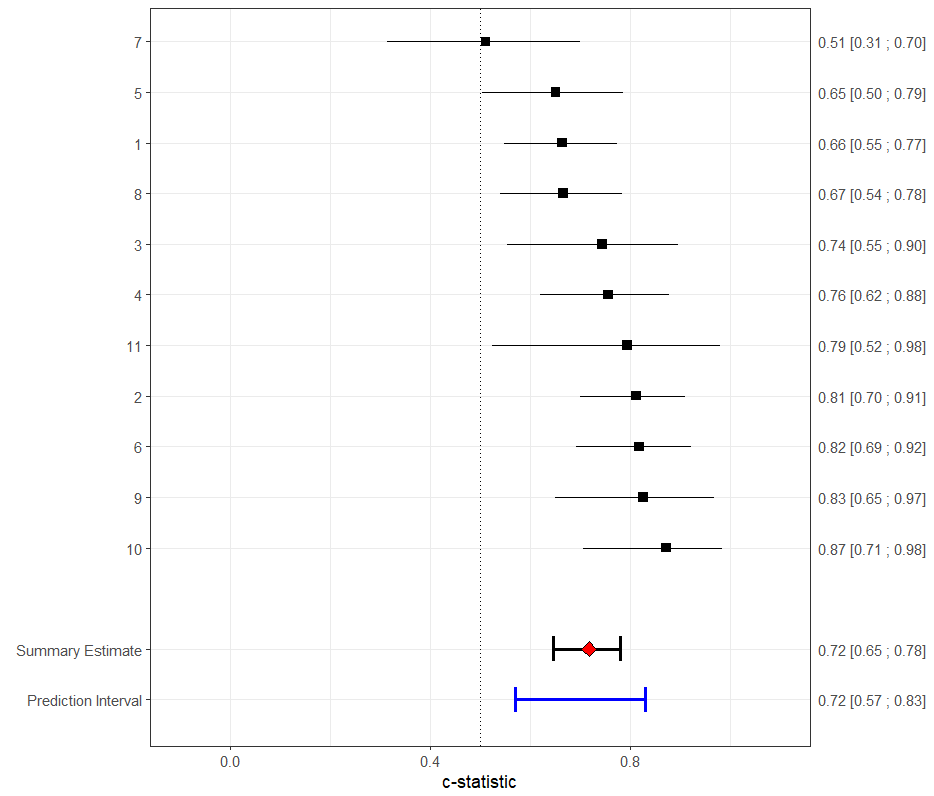


Note:

1.Meta-analysis was performed with the use of logit transformed c-statistics.

2.The prediction interval indicates a possible range of the c-statistic value in a new center.

**Figure S8**. Meta-analysis of hospital-specific slope estimates for the ACS-NSQIP model


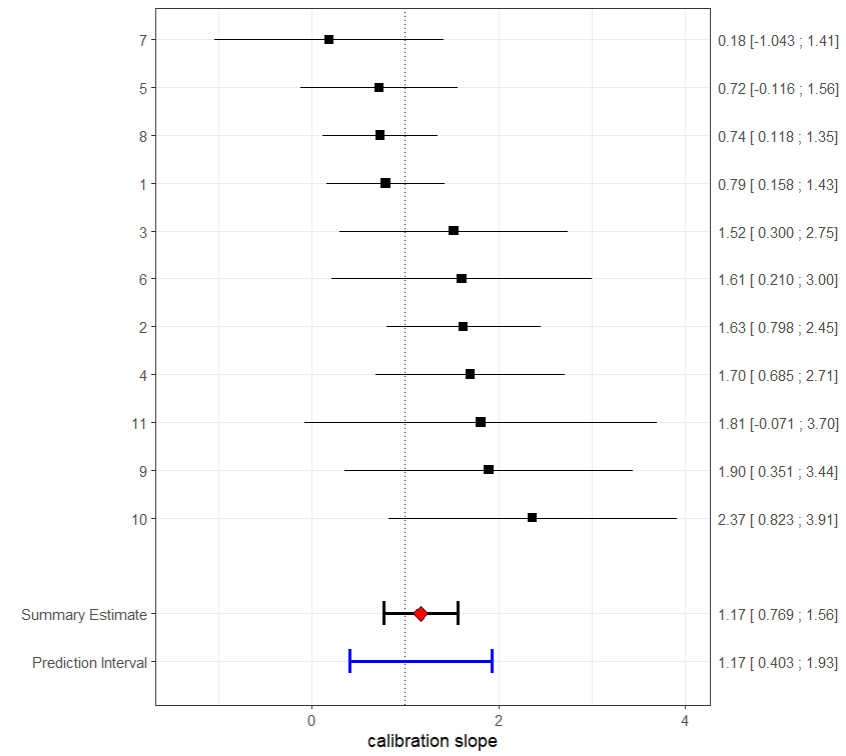


Note:

1.Meta-analysis was performed with the use of the original scale of the slopes.

2.The prediction interval indicates a possible range of the slope value in a new center.

**Figure S9**. Meta-analysis of hospital-specific intercept estimates for the ACS-NSQIP model


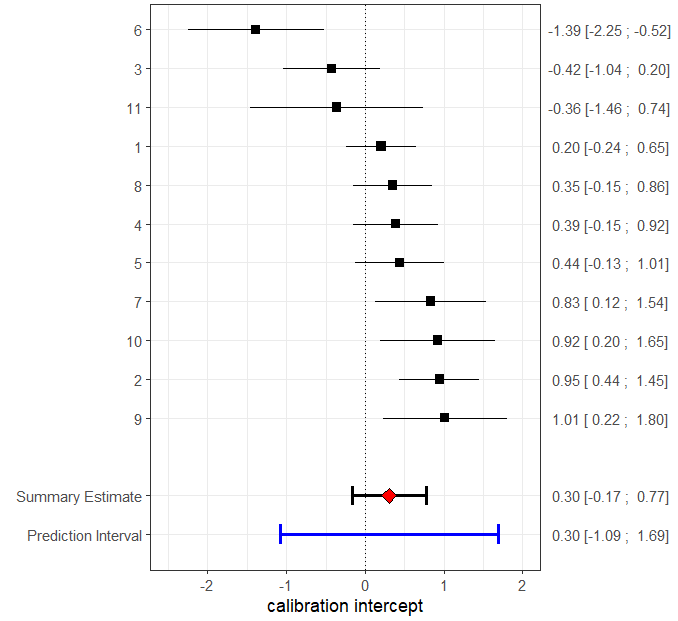


Note:

1.Meta-analysis was performed with the use of the original scale of the intercepts.

2.The prediction interval indicates a possible range of the intercept value in a new center.
